# Efficacy of three innovative bacterin vaccines against experimental infection with *Mycoplasma hyopneumoniae*

**DOI:** 10.1186/s13567-019-0709-0

**Published:** 2019-11-08

**Authors:** Anneleen Marguerite Filip Matthijs, Gaël Auray, Filip Boyen, Alexandra Schoos, Annelies Michiels, Obdulio García-Nicolás, Güliz Tuba Barut, Christophe Barnier-Quer, Virginie Jakob, Nicolas Collin, Bert Devriendt, Artur Summerfield, Freddy Haesebrouck, Dominiek Maes

**Affiliations:** 1 Department of Reproduction, Obstetrics and Herd Health, Faculty of Veterinary Medicine, Ghent University, Salisburylaan 133, 9820 Merelbeke, Belgium; 2 Institute of Virology and Immunology, Sensemattstrasse 293, 3147 Mittelhäusern, Switzerland; 3 Department of Infectious Diseases and Pathobiology, Vetsuisse Faculty, University of Bern, Länggassstrasse 120, 3012 Bern, Switzerland; 4 Department of Pathology, Bacteriology and Avian Diseases, Faculty of Veterinary Medicine, Ghent University, Salisburylaan 133, 9820 Merelbeke, Belgium; 5 Vaccine Formulation Laboratory, University of Lausanne, Chemin des Boveresses 155, 1066 Epalinges, Switzerland; 6 Laboratory of Veterinary Immunology, Department of Virology, Parasitology and Immunology, Faculty of Veterinary Medicine, Ghent University, Salisburylaan 133, 9820 Merelbeke, Belgium

## Abstract

New vaccine formulations that include novel strains of *Mycoplasma hyopneumoniae* and innovative adjuvants designed to induce cellular immunity could improve vaccine efficacy against this pathogen. The aim of this experimental study was to assess the efficacy of three experimental bacterin formulations based on *M. hyopneumoniae* field strain F7.2C which were able to induce cellular immunity. The formulations included a cationic liposome formulation with the Mincle receptor ligand trehalose 6,6-dibehenate (Lipo_DDA:TDB), a squalene-in-water emulsion with Toll-like receptor (TLR) ligands targeting TLR1/2, TLR7/8 and TLR9 (SWE_TLR), and a poly(lactic-*co*-glycolic acid) micro-particle formulation with the same TLR ligands (PLGA_TLR). Four groups of 12 *M. hyopneumoniae*-free piglets were primo- (day (D) 0; 39 days of age) and booster vaccinated (D14) intramuscularly with either one of the three experimental bacterin formulations or PBS. The pigs were endotracheally inoculated with a highly and low virulent *M. hyopneumoniae* strain on D28 and D29, respectively, and euthanized on D56. The main efficacy parameters were: respiratory disease score (RDS; daily), macroscopic lung lesion score (D56) and log copies *M. hyopneumoniae* DNA determined with qPCR on bronchoalveolar lavage (BAL) fluid (D42, D56). All formulations were able to reduce clinical symptoms, lung lesions and the *M. hyopneumoniae* DNA load in the lung, with formulation SWE_TLR being the most effective (RDS_D28–D56_ −61.90%, macroscopic lung lesions −88.38%, *M. hyopneumoniae* DNA load in BAL fluid (D42) −67.28%). Further experiments raised under field conditions are needed to confirm these results and to assess the effect of the vaccines on performance parameters.

## Introduction

*Mycoplasma hyopneumoniae* (*M. hyopneumoniae*) is the primary pathogen of enzootic pneumonia (EP) in pigs. This chronic respiratory disease is responsible for major economic losses in pig producing countries all over the world due to reduced performance and increased medication use [1, 2].

Together with biosecurity measurements, management practices and medication, commercial bacterin vaccines are used worldwide to control EP [1]. They are mostly constituted of inactivated whole cells of the J strain, a low virulent *M. hyopneumoniae* field strain isolated in the UK in the 1950s [3], and adjuvants such as aluminium hydroxide, carbopol, mineral oil or biodegradable oil [4]. Vaccination reduces clinical symptoms, lung lesions and performance losses [5, 6]. However, current commercial vaccines neither prevent colonisation of the pathogen, nor the development of clinical signs and lung lesions [7]. Also, their effect on disease transmission is only limited [8–10]. Moreover, the beneficial effects of vaccination are known to vary between herds [4], which may be partially due to pathogenic and antigenic differences between the strains circulating on the farms and the vaccine strain [11].

While serum antibodies are not correlated with protection against EP [12], the role of mucosal antibodies (immunoglobulin (Ig) A) is still unclear. According to Thacker et al. [13], mucosal IgA could prevent colonisation of the microorganism in the respiratory tract of the pig. Cell-mediated immune responses, more specifically T helper (Th) 1, Th17 and CD8^+^ T cell responses, are considered to play a major role in protective immunity against *M. hyopneumoniae* infections [13–16]. T helper 1 cells are considered to contribute to protection against *Mycoplasma* infections by activating macrophage killing [14], while Th17 cells protect the lung mucosa by elevating secretory IgA levels [17] and recruiting neutrophils for pathogen clearance [18]. CD8^+^ T cells, on the other hand, might dampen the excessive pro-inflammatory Th responses that induce lung lesions and clinical disease [19].

Research into novel vaccine formulations that may offer a better protection against *M. hyopneumoniae* is constantly ongoing. An overview of the different experimental *M. hyopneumoniae* vaccines already showed that most of them are based on recombinant proteins of *M. hyopneumoniae* and were evaluated in mice [4]. Merely a few of them were tested in challenge experiments in pigs. Also, none of these formulations were able to offer total protection or similar protection as the commercially available vaccines, despite their often promising immunizing properties [4, 20].

In a previous study [21], the safety and the immune responses of five innovative bacterin formulations were evaluated in pigs. All formulations were based on *M. hyopneumoniae* strain F7.2C, a highly virulent field strain shown to be antigenically different from the J strain [22, 23], and contained adjuvants specifically designed to promote cellular immunity. Three promising vaccine formulations were identified based on their ability to induce potent *M. hyopneumoniae*-specific T cell responses. These included a micro-particle and an oil-in-water formulation to deliver a cocktail of Toll-like receptor (TLR) 1/2, TLR9 and TLR7/8 ligands, and a cationic liposomal formulation to deliver a Mincle ligand. The liposomal formulation was able to induce strong Th1 and CD8^+^ T cell responses, while the oil-in-water formulation induced a strong Th1 response and a moderate CD8^+^ T cell response. The micro-particle formulation had the unique ability of inducing a potent Th17 response. Therefore, the aim of this study was to assess the protective efficacy of these three innovative bacterin formulations against experimental infection with two *M. hyopneumoniae* field strains. The main efficacy parameters were respiratory disease score (RDS), macroscopic lung lesion score and log copies *M. hyopneumoniae* DNA in bronchoalveolar lavage (BAL) fluid. Additionally, microscopic lung lesions, *M. hyopneumoniae*-specific local and systemic antibodies, *M. hyopneumoniae*-specific systemic T cell responses and cytokine responses in BAL fluid were assessed.

This study demonstrated the potential of innovative *M. hyopneumoniae* bacterin formulations and identified promising vaccine candidates for further exploration.

## Materials and methods

### Vaccines and adjuvants

Three adjuvant formulations were developed based on the association of particle-based delivery systems (liposomes, poly(lactic-*co*-glycolic acid) (PLGA) micro-particles and a squalene-in-water emulsion (SWE)) with different immune stimulators. These included the Mincle agonist trehalose 6,6-dibehenate (Avanti, Alabaster, AL, USA) and a combination of TLR ligands: TLR1/2 ligand Pam3Cys-SK4 (PAM; EMC Microcollections, Tübingen, Germany), TLR9 ligand CpG oligodeoxynucleotides SL03 (CpG; Eurofins Genomics, Les Ulis, France) and TLR7/8 ligand resiquimod (Chemdea, Ridgewood, NJ, USA). The selection of the TLR ligands was based on their ability to activate porcine antigen presenting cells [24–26].

Trehalose 6,6-dibehenate (TDB) was combined with dimethyl dioctadecylammonium (DDA) bromide with the thin lipid film method [27] and followed by extrusion to form the cationic liposome formulation Lipo_DDA:TDB. Cationic poly(lactic-*co*-glycolic acid) micro-particles (combined to ethylaminoethyl-dextran) were produced with the double emulsion (W/O/W) methods [28]. Pam3Cys-SK4 and resiquimod were encapsulated into the particles and CpG was subsequently adsorbed onto their surface for the PLGA_TLR formulation. The oil-in-water formulation SWE_TLR was obtained by mixing SWE (a squalene-based formulation developed and produced by the Vaccine Formulation Laboratory, and composed of 3.9% (w/v) squalene, 0.5% (w/v) Tween 80 and 0.5% (w/v) Span 85 [29]) with the immune stimulators PAM, CpG and resiquimod.

The vaccine strain *M. hyopneumoniae* F7.2C was grown in modified Friis medium [30] for 5 days at 35 ± 2 °C. The culture, containing 5 × 10^8^ colour changing units (CCU)/mL, was inactivated by incubation with 4 mM binary ethyleneimine (BEI) under agitation at 35 ± 2 °C for 24 h. Subsequently, the BEI was neutralised by incubating the inactivated culture with 4 mM sodium thiosulfate under agitation at 35 ± 2 °C for 24 h. Inactivated bacteria were pelleted at 15 000 *g* for 40 min at 4 °C and washed three times in 50 mL sterile phosphate buffered saline (PBS). The final pellet was resuspended in sterile PBS and mixed with the different adjuvants. The composition of each experimental vaccine is given in Table 1.

**Table 1:** **Composition of the experimental** ***M. hyopneumoniae*** **bacterins and their route of administration**

Vaccine formulation	Dose (mL)	Delivery system	Immune-stimulators (µg/dose)	Antigen dose (CCU/dose)	Administration route (primo and booster)
Lipo_DDA:TDB	2	DDA liposomes	TDB (500)	10^9^	IM
PLGA_TLR		PLGA micro-particles (combined to ethylaminoethyl-dextran)	Pam3Cys-SK4/CpG ODN SL03/resiquimod (80/80/80)		
SWE_TLR		Squalene-in-water emulsion			

CCU: colour changing units, IM: intramuscular, PLGA: poly(lactic-*co*-glycolic acid), DDA: dimethyl dioctadecylammonium, TDB: trehalose 6,6′-dibehenate.

### Study animals and experimental design

The study was performed after approval by the Ethical Committee for Animal Experiments of the Faculty of Veterinary Medicine, Ghent University (approval number EC2017/120). Fifty-three *M. hyopneumoniae*-free Rattlerlow-Seghers piglets (RA-SE Genetics NV, Ooigem, Belgium) were enrolled in the study. All animals originated from a herd that has been *M. hyopneumoniae*-free for many years based on repeated serological testing, nested PCR testing on tracheobronchial swabs, and absence of clinical signs and pneumonia lesions at slaughter. The piglets arrived at the experimental facilities of the Faculty of Veterinary Medicine, Ghent University, Belgium at 32 days of age. They were housed in stables with absolute air filters for impending particles (HEPA U15) on both incoming and outgoing ventilation shafts and fed ad libitum with a non-antimicrobial-supplemented diet. On the day of arrival (D-7), the piglets were randomly allocated into three vaccination groups and a PBS-injected control group (PCG) of 12 piglets each. Five pigs were included as a non-challenge control group (NCG). After an acclimatization period of 1 week, the pigs of the vaccination groups were prime-boost vaccinated intramuscularly (IM) at day (D) 0 and D14 of the study with 2 mL of experimental bacterin. The pigs of the PCG and NCG received 2 mL sterile PBS IM at both vaccination days. The pigs of the vaccinated groups and the PCG were experimentally infected by endotracheal inoculation of the highly virulent *M. hyopneumoniae* strain F7.2C (7 mL culture medium containing 10^7^ CCU/mL) on D28 and the low virulent strain F1.12A (7 mL culture medium containing 10^7^ CCU/mL) on D29 [31]. The pigs of the NCG were endotracheally inoculated with sterile culture medium on both challenge days. For the inoculations, the pigs were anesthetized by administering 0.22 mL/kg body weight of a mixture of Zoletil 100^®^ (Virbac, Louvain la Neuve, Belgium) and Xyl-M^®^ 2% (VMD, Arendonk, Belgium) IM. Four weeks after challenge infection (D56), all pigs were euthanized by exsanguination after deep anaesthesia with 0.3 mL/kg of the same mixture injected IM.

### Clinical and performance parameters

The pigs were observed daily between 8 and 10 a.m. for at least 20 min from D-6 until D56 of the study. For each pig, remarkable clinical findings (loss of appetite, diarrhoea, dyspnoea, depression, lameness) were recorded and the severity of coughing was assessed by means of a RDS [32]. The scoring could range from 0 to 6 with 0 = no coughing, 1 = mild coughing after an encouraged move, 2 = mild coughing in rest, 3 = moderate coughing after an encouraged move, 4 = moderate coughing in rest, 5 severe coughing after an encouraged move, 6 = severe coughing in rest.

On the day of primo-vaccination (D0), challenge (D28) and euthanasia, the pigs were weighed, and the average daily gain (ADG; g/pig/day) was calculated from D0–28, D28–56 and D0–56 [33].

### Macroscopic and microscopic lung lesions

At necropsy (D56), the lungs were removed and scored for macroscopic *Mycoplasma*-like lesions according to Hannan et al. [34]. The score could range from 0 (no lesions) to 35 (entire lung affected).

From each pig, samples from the left apical, cardiac and diaphragmatic lung lobe were collected for histopathological examination. If lesions were present, samples were taken from the border of the lesion. Each sample was scored for the degree of peribronchiolar and perivascular lymphohistiocytic infiltration, as well as nodule formation, using light microscopy. A score system ranging from 1 to 5 was used, with 1 = limited infiltration of macrophages and lymphocytes around bronchioles, with airways and alveolar spaces free of cellular exudates, 2 = light to moderate infiltrates with mild diffuse cellular exudates into airways, 3–4–5 = respectively mild, moderate and severe lesions characteristic of broncho-interstitial pneumonia, centred around bronchioles but extending to the interstitium, with lymphofollicular infiltration and mixed inflammatory cell exudates [35]. Scores 1 and 2 are considered to be not related to *M. hyopneumoniae*, while scores 3–5 are suggestive for an infection with *M. hyopneumoniae*.

The percentage of lung area occupied by air (% air) was assessed using an automated image analysis system (Leica Application Suite AF Lite (Diegem, Belgium) and ImageJ (Bethesda Softworks, Rockville, MD, USA) [31]).

### Quantitative PCR for *M. hyopneumoniae* DNA and routine bacteriological culture on bronchoalveolar lavage fluid

Two weeks after challenge infection (D42), bronchoalveolar lavage fluid was collected from each pig by inserting a catheter (Portex^®^ Dog Catheter with Female Luer Mount, Smiths Medical International Ltd., Kent, UK) in the trachea and flushing the lungs with 20 mL sterile PBS [31]. At necropsy (D56), BAL fluid was collected from the right lung by flushing the head bronchus with 20 mL sterile PBS as previously described [36]. Deoxyribonucleic acid was extracted from the BAL fluid using a commercial kit (DNeasy^®^ Blood & Tissue kit, Qiagen, Venlo, The Netherlands) and a quantitative PCR (qPCR) was performed according to Marois et al. [37] to measure the number of *M. hyopneumoniae* organisms. The threshold values were converted to the number of organisms using a tenfold dilution series of *M. hyopneumoniae* F7.2C DNA. Values below the highest dilution (1.50 × 10^1^/mL; 1.18 log copies/mL) were considered as negative.

From each pig, 10 µL of BAL fluid collected at D56 was inoculated on a Columbia agar supplemented with 5% sheep blood (Oxoid Limited, Hampshire, UK) with a *Staphylococcus pseudintermedius* streak [38]. The agar plates were incubated in a 5% CO_2_-enriched atmosphere at 35 ± 2 °C for 48 h to detect the presence of other respiratory bacteria.

### *M. hyopneumoniae*-specific antibody responses

Before primo-vaccination (D0), on the day of booster vaccination (D14), at challenge (D28), 2 weeks after challenge (D42) and at euthanasia (D56), serum samples were collected and the number of *M. hyopneumoniae* seropositive animals was determined with a commercial blocking ELISA (IDEIA™ *Mycoplasma hyopneumoniae* EIA kit, Oxoid Limited, Hampshire, UK) according to the manufacturer‘s instructions. Samples with optical density (OD) lower than 50% of the average OD of the buffer control were considered positive. Samples with OD-values equal or bigger than 50% of the average OD of the buffer control were considered negative.

*Mycoplasma hyopneumoniae*-specific IgG and IgA isotypes were measured in serum (diluted 1:200 and 1:100, respectively) with an in-house indirect ELISA using Tween 20-extracted *M. hyopneumoniae* antigens according to Matthijs et al. [21]. All samples were tested in duplicate. To relatively quantify the antibody levels, a standard curve was made using twofold serial dilutions of a positive reference serum corresponding to defined arbitrary units (1:800 and 1:200 dilution defined as 1 unit for IgG and IgA, respectively). Optical density values of the samples were interpolated from the standard curve using non-linear regression with least square fits in Graphpad Prism 8.0 (GraphPad Software Inc., San Diego, CA, USA).

*Mycoplasma hyopneumoniae*-specific IgA antibodies in BAL fluid collected 2 weeks after challenge infection (D42) and at euthanasia (D56) were measured with an in-house indirect ELISA as previously described [21]. The BAL fluid was tested undiluted and in duplicate. Antibody levels were also relatively quantified as described above using a standard curve made with positive BAL fluid (1:32 dilution defined as 1 unit). Animals with values higher than 0 arbitrary units were considered as positive, while animals with values equal to 0 arbitrary units were classified as negative.

### T cell assays

Shortly before challenge infection (D28) and at euthanasia (D56), blood samples were collected to assess *M. hyopneumoniae*-specific T cell responses according to Matthijs et al. [21]. Peripheral blood mononuclear cells (PBMCs) were isolated and restimulated in vitro overnight (18 h) with *M. hyopneumoniae* F7.2C bacterin. For the last 4 h, Brefeldin A was added to inhibit cytokine release and allow intracellular detection of cytokines. Subsequently, cells were harvested and the percentage of cytokine-producing T cells was measured by flow cytometry (FCM) using a 5-step 6-colour staining protocol. Briefly, following incubation with the LIVE/DEAD™ Fixable Aqua Dead Cell Stain Kit (Invitrogen™, ThermoFisher Scientific, Waltham, MA, USA), cells were incubated with anti-CD4 (clone 74-12-4, Southern Biotech, Birmingham, AL, USA) and anti-CD8β (clone PG164A, WSU, Pullman, WA, USA) antibodies and then with their corresponding secondary antibodies anti-mouse IgG2b AlexaFluor 488 (Molecular Probes, Eugene, OR, USA) and anti-mouse IgG2a PE-Cy7 (Abcam, Cambridge, UK). After fixation and permeabilization of the cells with the BD Cytofix/Cytoperm™ Fixation/Permeabilization Solution kit (Becton–Dickinson, Franklin Lakes, NJ, USA), intracellular cytokines were stained with directly coupled anti-human TNF-α AlexaFluor 647 (clone MAb11, BioLegend, San Diego, CA, USA), anti-pig IFN-γ PerCP-Cy5.5 (clone P2G10, Becton–Dickinson) and anti-human IL-17A PE (clone SCPL1362, Becton–Dickinson). All samples were acquired on a CytoFLEX flow cytometer (Beckman Coulter, Brea, CA, USA) and the analysis performed with the FlowJo™ software (Tree Star Inc., Ashland, OR, USA). For each animal, samples were restimulated in triplicate cultures and analysed separately. To define whether an animal had *M. hyopneumoniae*-specific circulating cells, a threshold value was calculated as the mean % of cytokine-producing cells_all control animals_ + 3*SD_all control animals_ for D28, and as the mean % of cytokine-producing cells_NCG_ + 3*SD_NCG_ for D56. Animals with values above the threshold were identified as positive, while animals with values equal to or below the threshold were classified as negative.

### Cytokines in BAL fluid

The BAL fluid collected at D42 and D56 was tested undiluted for the presence of IL-1β (Porcine IL-1 beta/IL-1F2 DuoSet^®^ ELISA, R&D Systems, Minneapolis, MN, USA), IL-6 (Porcine IL-6 DuoSet^®^ ELISA, R&D Systems, Minneapolis, MN, USA), IFN-γ (Swine IFN-γ Antibody Pair, Invitrogen™, ThermoFisher Scientific) and TNF-α (Swine TNF-α CytoSet™, Invitrogen™, ThermoFisher Scientific) using a sandwich ELISA according to the manufacturer‘s instructions. The OD-values were converted to cytokine levels by means of a standard curve.

### Statistical analyses

The RDS data was averaged for the periods D-6 until D56, D-6 until D27 and D28 until D56, and analysed using a repeated measures ANOVA. Pairwise comparisons between groups were obtained with Scheffé’s post hoc test. The T cell data was analysed using a one-way ANOVA with Tukey–Kramer’s post hoc test for pairwise comparisons. The parameters ADG, macroscopic lung lesions, microscopic lung lesions, % air, log copies *M. hyopneumoniae* DNA in BAL fluid, *M. hyopneumoniae*-specific IgG, *M. hyopneumoniae*-specific IgA (serum, BAL fluid), IL-1, IL-6, IFN-γ and TNF-α were not normally distributed according to the Shapiro–Wilk’s test, and were analysed using a Kruskal–Wallis ANOVA followed by Dunn’s post hoc test. Adjusted *P* values were computed to account for multiple comparisons, except for Scheffé’s post hoc test, which is already quite conservative [39]. The NCG was not included in the statistical analyses as this group only served as a sentinel group. Statistical analyses of efficacy parameters were conducted in SPSS 24 for Windows (IBM, Armonk, NY, USA). Immune response parameters were analysed using GraphPad Prism 8.0 (GraphPad Software Inc., San Diego, CA, USA). Statistical results were considered significant when *P *≤ 0.05.

## Results

### Clinical and performance parameters

General health, severity of coughing (daily RDS) and ADG of each piglet were closely monitored during the entire study. One piglet from the PCG died during anaesthesia on D28. This piglet was excluded from the RDS and ADG analyses. On D42, one piglet from the Lipo_DDA:TDB group showed severe abdominal breathing after blood sampling. Therefore, BAL fluid was not collected from that animal on D42.

**Figure 1:**
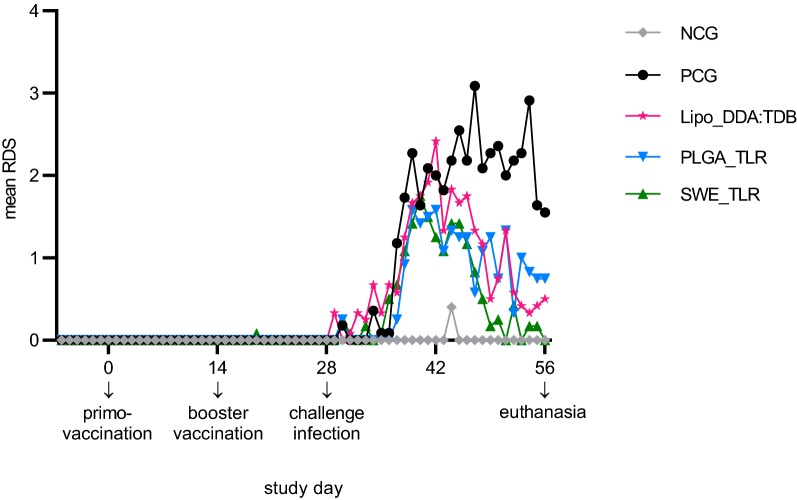
**Mean respiratory disease score for each group during the entire study.** NCG: non-challenge control group (PBS-injected, non-challenge infected), PCG: PBS-injected control group (PBS-injected, challenge infected), RDS: respiratory disease score.

None of the non-challenge control animals coughed, except for one pig on D44 (score 2, mild coughing in rest). An increase in the mean RDS was first observed in group Lipo_DDA:TDB from 6 to 8 days post challenge (DPC) onwards, followed by groups SWE_TLR, PCG and PLGA_TLR, respectively from 8, 9 and 10 DPC onwards. After several days, coughing decreased in the vaccinated groups, while it continued at a high level in the PCG until the end of the study (Figure 1). After challenge infection (D28–56), formulation SWE_TLR induced the highest reduction in mean RDS compared to the PCG (61.90%), followed by formulations PLGA_TLR and Lipo_DDA:TDB (50.34% and 38.78%, respectively). However, the reduction of coughing was only statistically significant for group SWE_TLR (*P *≤ 0.05; Table 2).

**Table 2:** **Overview of the efficacy data**

Parameter	Study day	NCG	PCG	Lipo_DDA:TDB	PLGA_TLR	SWE_TLR	*P* value
RDS	−6 until 56	0.01 ± 0.01	0.68 ± 0.32^b^	0.42 ± 0.33^ab^	0.33 ± 0.35^ab^	0.26 ± 0.15^a^	0.011
	−6 until 27	0.00 ± 0.00	0.00 ± 0.00^a^	0.00 ± 0.00^a^	0.00 ± 0.00^a^	0.00 ± 0.01^a^	0.415
	28 until 56	0.01 ± 0.03	1.47 ± 0.69^b^	0.90 ± 0.73^ab^	0.73 ± 0.77^ab^	0.56 ± 0.33^a^	0.011
ADG in g/pig/d	0 until 56	691 ± 69	655 ± 109^a^	610 ± 90^a^	628 ± 97^a^	688 ± 49^a^	0.065
	0 until 28	620 ± 47	592 ± 104^a^	560 ± 104^a^	586 ± 106^a^	581 ± 70^a^	0.526
	28 until 56	762 ± 98	739 ± 141^ab^	660 ± 96^a^	669 ± 127^a^	795 ± 69^b^	0.004
Number of animals with macroscopic lung lesions (% positive animals)	56	0/5 (0.00)	11/11 (100.00)	7/12 (58.33)	7/12 (58.33)	7/12 (58.33)	–
Macroscopic lung lesion score	56	0.00 ± 0.00	7.57 ± 5.18^b^	2.28 ± 3.55^a^	1.43 ± 2.31^a^	0.88 ± 1.01^a^	0.002
Microscopic lung lesion score	56	1.13 ± 0.18	3.73 ± 0.94^b^	2.11 ± 0.89^a^	2.22 ± 0.87^a^	1.78 ± 0.57^a^	< 0.001
Percentage of lung area occupied by air	56	39.11 ± 6.26	23.38 ± 6.43^b^	37.57 ± 5.99^a^	34.82 ± 4.28^ab^	42.15 ± 7.98^a^	< 0.001
Number of animals positive for *M. hyopneumoniae* in BAL fluid (% positive animals)	42	0/5	11/11 (100.00)	9/11 (81.82)	9/12 (75.00)	6/12 (50.00)	–
	56	0/5	8/11 (72.73)	5/12 (41.67)	6/12 (50.00)	4/12 (33.33)	–
Log copies *M. hyopneumoniae* DNA/mL BAL fluid	42	0.00 ± 0.00	3.82 ± 0.71^b^	2.50 ± 1.18^ab^	2.20 ± 1.38^a^	1.25 ± 1.28^a^	< 0.001
	56	0.00 ± 0.00	1.74 ± 0.94^a^	1.24 ± 1.34^a^	1.76 ± 1.68^a^	0.63 ± 0.74^a^	0.082

Pigs were prime-boost vaccinated on D0 and D14 with three different experimental *M. hyopneumoniae* bacterins (Lipo_DDA:TDB, PLGA_TLR, SWE_TLR), challenge infected on D28–29 and euthanized on D56. For the parameters ADG, macroscopic lung lesion score, microscopic lung lesion score, % air and number of *M. hyopneumoniae* organisms in BAL fluid, a Kruskal–Wallis ANOVA was performed to determine statistical differences between the groups on each time point. For the parameter RDS, a repeated measurements ANOVA with Scheffé’s post hoc test was performed. The NCG was not included in the statistical analyses. Groups that have no superscript in common are significantly different from each other (*P* ≤ 0.05). All results are expressed as mean ± SD, unless otherwise stated.

NCG: non-challenge control group (non-vaccinated, non-challenge infected), PCG: PBS-injected control group (non-vaccinated, challenge infected), RDS: respiratory disease score, ADG: average daily gain, BAL: bronchoalveolar lavage.

For each group, the mean ADG from D0–56, D0–28 and D28–56 is shown in Table 2. After challenge infection (D28–56), ADG from group SWE_TLR was significantly higher compared to the other vaccinated groups (*P *≤ 0.05).

### Macroscopic and microscopic lung lesions

At euthanasia (D56), lungs were collected and scored for macroscopic and microscopic lung lesions. The percentage of lung area occupied by air was measured using image analysis. The results are presented in Table 2. Macroscopic lung lesions were observed in all animals from the PCG, and in 7/12 animals from each of the vaccinated groups. In the NCG, none of the animals either had macroscopic lung lesions, nor microscopic lung lesion scores higher than 2. All vaccinated groups had a significantly lower macroscopic lesion score compared to the PCG (*P *≤ 0.05). Formulation SWE_TLR induced the highest reduction in macroscopic lung lesions compared to the PCG (88.38%), while formulations PLGA_TLR and Lipo_DDA:TDB reduced macroscopic lung lesions with 81.11% and 69.88%, respectively. All three formulations significantly reduced microscopic lung lesions (*P *≤ 0.05), and the highest reduction was again observed in group SWE_TLR. Groups Lipo_DDA:TDB and SWE_TLR had a significantly higher percentage of lung area occupied by air compared to the PCG (*P *≤ 0.05).

### Quantitative PCR for *M. hyopneumoniae* DNA and routine bacteriological culture on bronchoalveolar lavage fluid

The number of animals positive for *M. hyopneumoniae* DNA in BAL fluid and mean log copies *M. hyopneumoniae* DNA in BAL fluid are shown for each group in Table 2. Two weeks after challenge infection (D42), significantly lower numbers of *M. hyopneumoniae* organisms were detected in BAL fluid from groups PLGA_TLR and SWE_TLR compared to the PCG (*P *≤ 0.05). The reduction in log copies *M. hyopneumoniae* DNA was 42.41% and 67.28%, respectively. Formulation Lipo_DDA:TDB reduced the number of *M. hyopneumoniae* organisms in BAL fluid with 34.55% compared to the PCG, but this reduction was not statistically significant (*P *> 0.05). At euthanasia (D56), all groups had a lower number of *M. hyopneumoniae* DNA in BAL fluid compared to D42 and no significant differences were observed between the groups (*P *> 0.05).

No other respiratory bacteria were detected after inoculating the BAL samples on Columbia blood agar plates.

### *M. hyopneumoniae*-specific antibody responses

According to the commercial blocking ELISA (Additional file 1), all animals from group Lipo_DDA:TDB were positive for *M. hyopneumoniae*-specific antibodies in serum 2 weeks after booster vaccination (D28). In groups PLGA_TLR and SWE_TLR, 5/12 and 11/12 pigs seroconverted, respectively. Two weeks after challenge infection (D42), all pigs from the vaccinated groups were seropositive, together with 7/11 pigs from the PCG. At euthanasia (D56), all pigs from the vaccinated groups and PCG were seropositive. The pigs form the NCG remained serologically negative for *M. hyopneumoniae* during the entire study.

**Figure 2:**
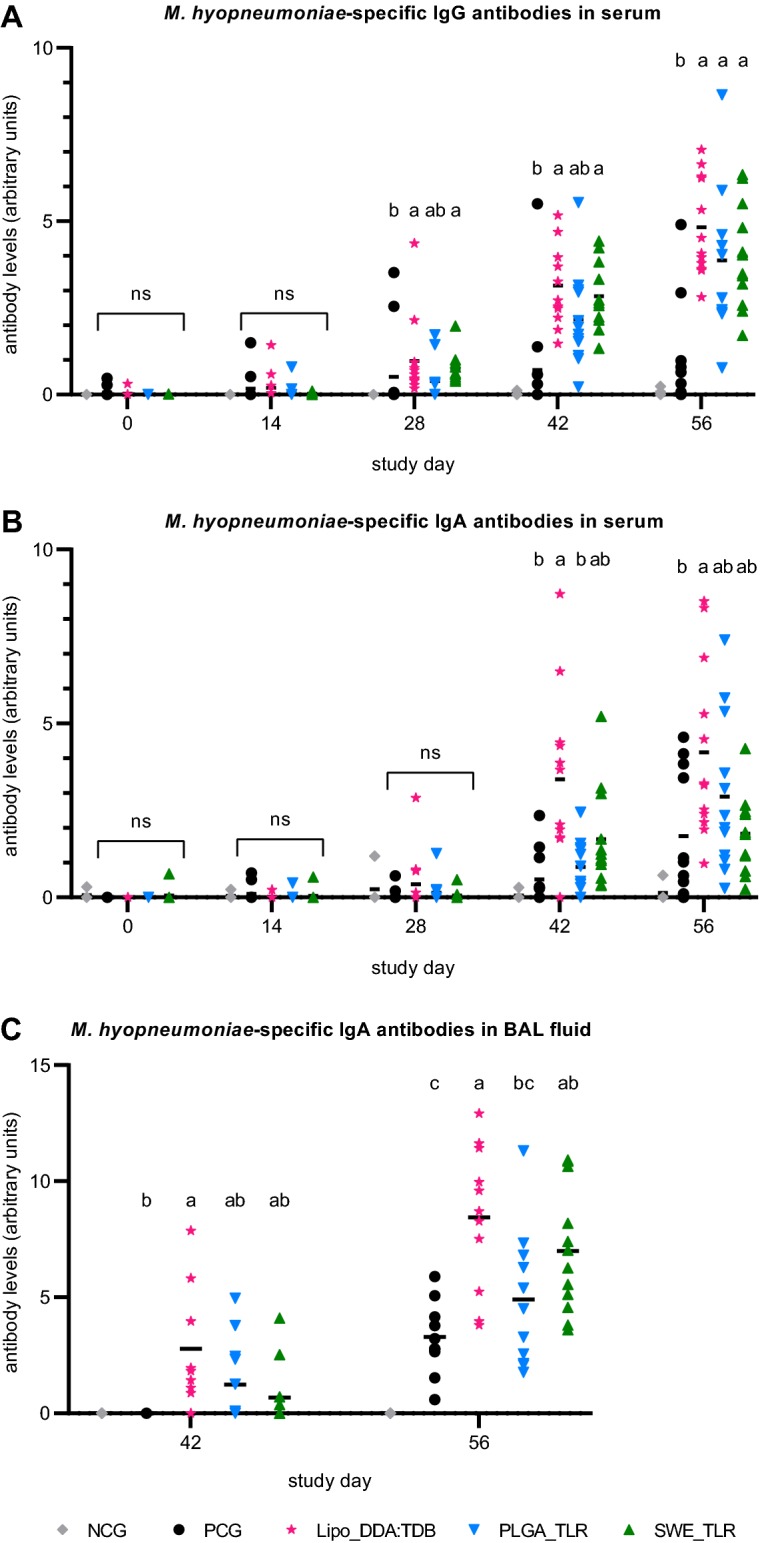
**Antibody levels following vaccination and challenge infection.** Pigs were prime-boost vaccinated on D0 and D14 with three different experimental *M. hyopneumoniae* bacterins (Lipo_DDA:TDB, PLGA_TLR, SWE_TLR), challenge infected on D28–29 and euthanized on D56. *M. hyopneumoniae*-specific IgG (**A**) and IgA (**B**) antibodies in serum and *M. hyopneumoniae*-specific IgA in BAL fluid (**C**) were determined by indirect ELISA. Individual animals are shown. For each time point, significance was calculated using a Kruskal–Wallis ANOVA. The NCG was not included in the statistical analyses. Groups that have no superscript in common are significantly different from each other (*P* ≤ 0.05). NCG, non-challenge control group (PBS-injected, non-challenge infected); PCG, PBS-injected control group (PBS-injected, challenge infected).

*Mycoplasma hyopneumoniae*-specific IgG and IgA levels in serum and *M. hyopneumoniae*-specific IgA levels in BAL fluid were quantified using an in-house indirect ELISA with positive reference serum or BAL fluid as a standard (Figures 2A–C). Two weeks after booster vaccination (D28), formulations Lipo_DDA:TDB and SWE_TLR induced a significant *M. hyopneumoniae*-specific IgG response (*P *≤ 0.05). Two weeks after challenge infection (D42) and at euthanasia (D56), all vaccinated groups had higher *M. hyopneumoniae*-specific IgG levels compared to the PCG. This was statistically significant for all vaccinated groups (*P *≤ 0.05) except group PLGA_TLR on D42 (Figure 2A).

Two weeks after primo-vaccination (D14) and 2 weeks after booster vaccination (D28), none of the vaccine formulations induced a significant *M. hyopneumoniae*-specific IgA response in serum (*P* > 0.05). Nevertheless, 2 weeks after challenge infection (D42), group Lipo_DDA:TDB showed a significantly higher *M. hyopneumoniae*-specific IgA response compared to the PCG and group PLGA_TLR (*P *≤ 0.05). Also, at euthanasia (D56), group Lipo_DDA:TDB had a significantly higher level of *M. hyopneumoniae*-specific IgA in serum compared to the PCG (*P *≤ 0.05; Figure 2B).

According to the in-house IgA ELISA on BAL fluid, respectively 9/11, 6/12 and 5/12 animals from groups Lipo_DDA:TDB, PLGA_TLR and SWE_TLR had *M. hyopneumoniae*-specific IgA antibodies in BAL fluid collected 2 weeks after challenge (D42). At euthanasia (D56), all animals from the vaccinated groups and the PCG were positive for *M. hyopneumoniae*-specific IgA. No IgA antibodies were detected in BAL fluid from the non-challenge control animals on both sampling days (Additional file 1). Two weeks after challenge infection (D42), group Lipo_DDA:TDB showed a significantly higher *M. hyopneumoniae*-specific IgA response in BAL fluid compared to the PCG (*P *≤ 0.05). At euthanasia (D56), groups Lipo_DDA:TDB and SWE_TLR had significantly higher *M. hyopneumoniae*-specific IgA levels in BAL fluid compared to the PCG, and group Lipo_DDA:TDB was also significantly higher than group PLGA_TLR (*P *≤ 0.05; Figure 2C).

### T cell assays

**Figure 3:**
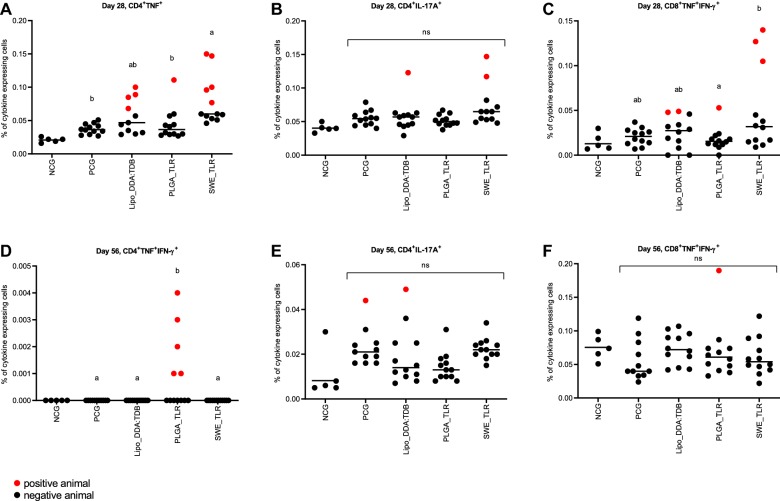
***M. hyopneumoniae*-specific T cell responses following vaccination and challenge infection.** Pigs were prime-boost vaccinated on D0 and D14 with three different experimental *M. hyopneumoniae* bacterins (Lipo_DDA:TDB, PLGA_TLR, SWE_TLR), challenge infected on D28–29 and euthanized on D56. *M. hyopneumoniae*-specific T cells were determined by in vitro restimulation of PBMC followed by intracellular cytokine staining and multicolour flow cytometry. Following doublet exclusion, live cells were gated and the percentage of TNF^+^ CD4^+^, IFNγ^+^TNF^+^ double positive CD4^+^ and CD8β^+^ T cells, and IL-17A^+^ CD4^+^ T cells was determined. The mean values obtained from triplicate cultures for individual animals are shown. Positive animals are marked in red (defined as being above the mean + 3*SD of all control animals on D28 and being above the mean + 3*SD of the NCG on D56). The horizontal line in each group represents the group mean. For each time point, significance between groups was calculated using one-way ANOVA followed by Tukey–Kramer’s test. The NCG was not included in the statistical analyses. Groups that have no superscript in common are significantly different from each other (*P* ≤ 0.05). NCG, non-challenge control group (PBS-injected, non-challenge infected); PCG, PBS-injected control group (PBS-injected, challenge infected); PBMC, peripheral blood mononuclear cells.

The results of the *M. hyopneumoniae*-specific T cell responses detected in the blood 2 weeks after booster vaccination (D28) are presented in Figures 3A–C. Group SWE_TLR had a significantly higher percentage of TNF-producing CD4^+^ (Th1) cells compared to the PCG and group PLGA_TLR (*P *≤ 0.05). However, in this group only five animals were above the threshold. In the Lipo_DDA:TDB group four animals and in group PLGA_TLR one animal were above the cut-off value (Figure 3A). For the CD4^+^ IL-17A^+^ (Th17) cells, one pig from group Lipo_DDA:TDB and two pigs from group SWE_TLR were above the cut-off (Figure 3B). Two pigs from group Lipo_DDA:TDB, 1 pig from group PLGA_TLR and three pigs from group SWE_TLR showed circulating CD8^+^ TNF^+^IFN-γ^+^ T cells at the time of sampling. The percentage CD8^+^ TNF^+^IFN-γ^+^ T cells in group SWE_TLR was significantly higher compared to the PLGA_TLR group (*P *≤ 0.05; Figure 3C).

Four weeks after challenge (D56), group PLGA_TLR had a significantly higher percentage CD4^+^ TNF^+^IFN-γ^+^ (Th1) cells compared to the PCG and the other two vaccinated groups (*P *≤ 0.05), although merely five animals appeared to have such cells in the blood (Figure 3D). In the blood of one pig from the PCG and 1 pig of the Lipo_DDA:TDB group CD4^+^ IL-17A^+^ T cells were detected (Figure 3E). Only in the PLGA_TLR group one animal had CD8^+^ TNF^+^IFN-γ^+^ T cells above the defined threshold (Figure 3F).

### Cytokines in BAL fluid

**Figure 4:**
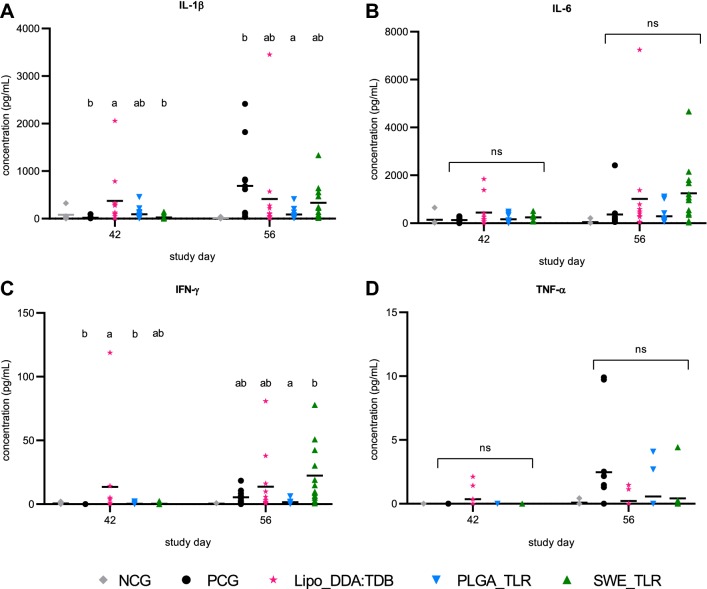
**Cytokine concentrations (pg/mL) in bronchoalveolar lavage fluid.** Pigs were prime-boost vaccinated on D0 and D14 with three different experimental *M. hyopneumoniae* bacterins (Lipo_DDA:TDB, PLGA_TLR, SWE_TLR), challenge infected on D28–29 and euthanized on D56. IL-1β (**A**), IL-6 (**B**), IFN-γ (**C**) and TNF-α (**D**) concentrations in BAL fluid were measured using commercial sandwich ELISAs. For each time point, significance was calculated using a Kruskal–Wallis ANOVA. The NCG was not included in the statistical analyses. Groups that have no superscript in common are significantly different from each other (*P* ≤ 0.05). NCG, non-challenge control group (PBS-injected, non-challenge infected), PCG, PBS-injected control group (PBS-injected, challenge infected), BAL, bronchoalveolar lavage.

The concentrations of IL-1β, IL-6, IFN-γ and TNF-α in BAL fluid collected on D42 and D56 are presented for each group in Figures 4A–D. Two weeks after challenge infection (D42), the IL-1β concentration in BAL fluid was significantly higher in group Lipo_DDA:TDB compared to the PCG and group SWE_TLR (*P *≤ 0.05). At euthanasia (D56), group PLGA_TLR had a significantly lower IL-1β concentration compared to the PCG (*P *≤ 0.05; Figure 4A).

Only in BAL fluid from group Lipo_DDA:TDB a significant level of IFN-γ was detected on D42 (*P *≤ 0.05). At euthanasia (D56), group SWE-TLR had a significantly higher IFN-γ concentration compared to group PLGA_TLR (*P *≤ 0.05; Figure 4C).

Regarding IL-6 and TNF-α, no statistically significant differences were observed between the groups on both time points (*P* > 0.05; Figures 4B and D).

## Discussion

The present study assessed the protective efficacy of three innovative *M. hyopneumoniae* bacterin formulations in a porcine experimental challenge model. The pigs were challenge infected with two *M. hyopneumoniae* field strains according to Michiels et al. [31]. These included strain F7.2C (the vaccine strain) and strain F1.12A, which were shown to differ from each other on a genomic [40], proteomic [23] and pathogenic [22] level. Challenge infection with two genetically different field strains might improve extrapolation to the field situation compared to experimental infection with only one strain, as the study from Michiels et al. demonstrated that most pigs were simultaneously infected with two or more genetically different *M. hyopneumoniae* strains under field conditions [41]. All animals from the PCG (PBS-injected, challenge infected) developed lung lesions, were positive for *M. hyopneumoniae* in BAL fluid and seroconverted, indicating that the challenge infection was successful. The values for the RDS and lung lesion scores in the PCG were comparable with those found in previous experimental studies using the same challenge model [31, 42].

According to the in-house serum ELISAs, some animals appeared to have *M. hyopneumoniae*-specific antibodies before vaccination and/or challenge infection (animals with values higher than 0 arbitrary units; Figure 2). These animals most likely tested false positive due to non-specific binding, since the study animals were obtained from a *M. hyopneumoniae*-free farm. Moreover, the particular pigs tested negative in the commercial blocking ELISA on those time points. While the commercial kit uses a highly specific monoclonal antibody against a conserved epitope of the *M. hyopneumoniae* 74 kDa protein, our in-house indirect ELISA is based on Tween 20-extracted proteins of *M. hyopneumoniae*. Such crude antigen preparations more easily allow cross-reactions with antibodies against the closely related *M. flocculare*, a commonly occurring commensal in the respiratory tract of pigs [43]. However, an additional *M. flocculare* ELISA to confirm the presence of such antibodies was not performed. In consequence, it remains uncertain whether all obtained in-house serum ELISA results are purely due to a *M. hyopneumoniae* response.

All three vaccine formulations were able to reduce clinical signs, macroscopic lung lesions and histopathological lung lesions, with formulation SWE_TLR being the most effective (RDS −61.9%, macroscopic lung lesions −88.4%, log copies *M. hyopneumoniae* DNA in BAL fluid −67.3%). The improvements obtained with formulation SWE_TLR seemed similar to or sometimes even better (adding up to 40% and 58% to the reduction of macroscopic lung lesions and log copies *M. hyopneumoniae* DNA, respectively) than the results obtained with commercial *M. hyopneumoniae* bacterins under experimental conditions [11, 31, 44, 45]. However, due to differences in the experimental settings (i.e. age of vaccination, challenge strains, one-shot vs. two-shot vaccination), comparisons with other trials remain speculative. In order to properly compare the protective efficacy of the experimental vaccines with the protection levels induced by commercial vaccines, a commercial two-shot vaccine should have been included in the experimental design. Interestingly, the protective efficacy of the highly virulent F7.2C strain formulated as a bacterin in combination with an aqueous adjuvant was assessed in a previous study [11]. However, in that study, the experimental vaccine formulation did not offer significant protection against experimental infection. This might be explained by the use of a less potent adjuvant and/or the lower antigen load (7.7 log10 CCU/mL) of the vaccine. Next to that, it should be mentioned that the vaccination was partially homologous to the challenge infection in this study, as strain F7.2C was used to construct the vaccines and was also one of the two challenge strains. One might suggest that this could result in better protection compared to challenge with strains different from the vaccine. Nevertheless, Villarreal et al. showed that vaccination with a bacterin homologous to the strain used for challenge infection did not result in an increased protection when compared to bacterins containing genetically heterologous strains [11].

Two weeks after challenge infection, groups SWE_TLR and PLGA_TLR had significantly lower numbers of *M. hyopneumoniae* organisms in BAL fluid compared to the PCG, indicating a lower shedding of *M. hyopneumoniae* in vaccinated pigs. However, like the current commercial vaccines, the experimental vaccine formulations from this study could not prevent colonization of the pathogen in the respiratory tract of the pigs.

Group SWE_TLR was the only group that had a higher ADG from the day of challenge until euthanasia. However, these findings were not statistically significant, most likely due to the small number of animals included in the study, the high SD of this parameter and the rather short study period [11, 31]. Further research including more animals and raised under field conditions is needed to obtain more reliable data on the impact of these experimental vaccines on performance parameters such as ADG and feed conversion ratio (FCR).

In accordance with the results from a previous study [21], formulation Lipo_DDA:TDB was the most potent in inducing a serological IgG response. However, the reduction in lung lesions was the lowest in group Lipo_DDA:TDB, confirming once more that systemic antibodies do not correlate with protection against EP [12]. Mucosal IgA is considered important to control *M. hyopneumoniae* infection, as adherence of the bacteria to the cilia of respiratory epithelium is the first step in the pathogenesis [13]. In the study from Matthijs et al. [21], only one animal from group SWE_TLR had *M. hyopneumoniae*-specific IgA antibodies in BAL fluid 2 weeks after booster vaccination. In the present study, groups Lipo_DDA:TDB and SWE_TLR had significantly more *M. hyopneumoniae*-specific IgA in BAL fluid collected 4 weeks after challenge compared to the non-vaccinated animals. Similar observations were made in previous studies [12, 45]. This increase of specific IgA in BAL fluid from the vaccinated groups indicates an anamnestic immune response, and suggests that priming of the mucosal immune system is possible after parenteral vaccine administration. As T cells are required for isotype switching, it also confirms the priming of specific T helper cells by all three vaccines. Although the *M. hyopneumoniae*-specific IgA levels in BAL fluid are not completely in line with its levels in serum, it can however not be stated with certainty that all the detected antigen-specific IgA in BAL fluid is produced locally. As with the *M. hyopneumoniae*-specific serum antibodies, the formulation inducing the highest antigen-specific IgA levels after challenge infection did not offer the highest protection. This indicates that also other arms of the immune system play an important role in the protection against EP. It is also possible that mucosal IgA is only protective if induced pre-challenge. Clearly, developing an effective mucosal vaccine is required to address this question.

Two weeks after booster vaccination (D28), a significantly higher percentage of *M. hyopneumoniae*-specific Th1 cells was observed in group SWE_TLR. Some animals in group Lipo_DDA:TDB also appeared to have such cells in the blood circulation. These results are in accordance with the results from the study of Matthijs et al. [21], where formulations Lipo_DDA:TDB and SWE_TLR induced a stronger *M. hyopneumoniae*-specific circulating Th1 response 2 weeks after booster vaccination. Only a few vaccinated animals had *M. hyopneumoniae*-specific circulating Th17 and CD8^+^ TNF^+^IFN-γ^+^ T cells, while the study of Matthijs et al. [21] showed a significant Th17 response in group PLGA_TLR and moderate to strong CD8^+^ T cell responses in groups SWE_TLR and Lipo_DDA:TDB, respectively. However, this lack of detectable *M. hyopneumoniae*-specific T cells should not be interpreted as a lack of T cell priming, as the frequency of antigen-specific T cells circulating in the peripheral blood compartment is a very dynamic process and changes over time. Following the expansion of specific T cells observed during a recall response (booster vaccination or challenge for this experiment), the contraction phase corresponds to a huge decrease in the frequency of antigen-specific T cells before they become memory cells. Even though memory cells are still circulating in the peripheral blood, their frequency is low as most memory cells recirculate between lymphoid tissue and blood, migrate to peripheral sites or the bone marrow for long-term survival [46, 47]. It appears that in this study formulation Lipo_DDA:TDB induced less circulating specific T cells compared to the study of Matthijs et al. [21], which might also be due to the fact that this vaccine was only applied IM in this study, while it was applied intradermally and IM at primo-vaccination in the previous study. The change in administration route was necessary due to severe local reactions at the intradermal injection site [21]. Interestingly, the SWE_TLR formulation appeared the most capable of inducing cellular immunity detectable in the blood, and also offered the highest protection. Altogether, the data of this study support the hypothesis that cellular immunity is important for protection against EP.

Two weeks after challenge infection, group Lipo_DDA:TDB had very high IL-1β levels compared to the other groups. This group also had the highest RDS at that time point. Several studies have associated the excessive production of pro-inflammatory cytokines such as IL-1, IL-6 and TNF-α with the development of *M. hyopneumoniae*-induced pneumonia [48–50]. According to Marchioro et al. [36], vaccination might reduce lung damage by regulating the release of these pro-inflammatory cytokines. However, in this study vaccination did not strongly impact the concentration of pro-inflammatory cytokines in the BAL fluid.

In conclusion, all formulations were able to reduce clinical symptoms, macro- and microscopic lung lesions and the *M. hyopneumoniae* DNA load in the lung, with the oil-in-water formulation delivering a cocktail of TLR-ligands being the most effective. As the number of animals is limited in experimental infection studies, further research including more animals and raised under field conditions is needed to confirm the present results, and especially to assess the effects of the different vaccine formulations from this study on the reduction of performance losses (ADG, FCR) due to *M. hyopneumoniae* infections.

## Supplementary information

**Additional file 1. Results of the** ***M. hyopneumoniae*-specific antibodies measured at different time points in serum and in BAL fluid.** Pigs were prime-boost vaccinated on D0 and D14 with three different experimental *M. hyopneumoniae* bacterins (Lipo_DDA:TDB, PLGA_TLR, SWE_TLR), challenge infected on D28–29 and euthanized on D56. *M. hyopneumoniae*-specific antibodies were determined by the IDEIA™ *Mycoplasma hyopneumoniae* EIA kit (Oxoid Limited, Hampshire, UK) and by indirect in-house ELISAs. For the in-house ELISAs, NetOD-values were calculated by subtracting the OD-value of the blank from the OD-value of the sample. BAL, bronchoalveolar lavage; NCG, non-challenge control group (PBS-injected, non-challenge infected); PCG, PBS-injected control group (PBS-injected, challenge infected); OD, optical density.

## Abbreviations

ADG: average daily gain
BAL: bronchoalveolar lavage
CCU: colour changing units
c-di-AMP: cyclic diadenylate monophosphate
CpG: CpG oligodeoxynucleotides SL03
D: day
DDA: dimethyl dioctadecylammonium
EP: enzootic pneumonia
FCM: flow cytometry
FCR: feed conversion ratio
Ig: immunoglobulin
IM: intramuscular, intramuscularly
NCG: non-challenge control group
OD: optical density
PAM: Pam3Cys-SK4
PBS: phosphate buffered saline
PCG: PBS-injected group
PLGA: poly(lactic-*co*-glycolic acid)
SD: standard deviation
SWE: squalene-in-water emulsion
TDB: trehalose 6,6-dibehenate
Th: T helper
TLR: Toll-like receptor
