# Supplementary material for: Efficacy of three innovative bacterin vaccines against experimental infection with Mycoplasma hyopneumoniae
Source: Vet Res. 2019 Nov 8;50:91. doi: 10.1186/s13567-019-0709-0 (PMC6842239; doi:10.1186/s13567-019-0709-0)
Supplement: Supplementary file 1 — Additional file 1. Results of the M. hyopneumoniae-specific antibodies measured at different time points in serum and in BAL fluid. Pigs were prime-boost vaccinated on D0 and D14 with three different experimental M. hyopneumoniae bacterins (Lipo_DDA:TDB, PLGA_TLR, SWE_TLR), challenge infected on D28–29 and euthanized on D56. M. hyopneumoniae-specific antibodies were determined by the IDEIA™ Mycoplasma hyopneumoniae EIA kit (Oxoid Limited, Hampshire, UK) and by indirect in-house ELISAs. For the in-house ELISAs, NetOD-values were calculated by subtracting the OD-value of the blank from the OD-value of the sample. BAL, bronchoalveolar lavage; NCG, non-challenge control group (PBS-injected, non-challenge infected); PCG, PBS-injected control group (PBS-injected, challenge infected); OD, optical density. [file 13567_2019_709_MOESM1_ESM.docx]

| **Parameter** | **Study day** | **NCG** | **PCG** | **Lipo_DDA:TDB** | **PLGA_TLR** | **SWE_TLR** |
| --- | --- | --- | --- | --- | --- | --- |
| Number of *M. hyopneumoniae* seropositive animals (% positive animals) determined with the IDEIA™ blocking ELISA from Oxoid | 0 | 0/5 (0.00) | 0/12 (0.00) | 0/12 (0.00) | 0/12 (0.00) | 0/12 (0.00) |
|  | 14 | 0/5 (0.00) | 0/12 (0.00) | 0/12 (0.00) | 0/12 (0.00) | 0/12 (0.00) |
|  | 28 | 0/5 (0.00) | 0/12 (0.00) | 12/12 (100.00) | 5/12 (41.67) | 11/12 (91.67) |
|  | 42 | 0/5 (0.00) | 7/11 (63.64) | 12/12 (100.00) | 12/12 (100.00) | 12/12 (100.00) |
|  | 56 | 0/5 (0.00) | 11/11 (100.00) | 12/12 (100.00) | 12/12 (100.00) | 12/12 (100.00) |
| *M. hyopneumoniae-*specific antibodies in serum measured with the IDEIA™ blocking ELISA from Oxoid (mean OD-values ± SD) | 0 | 0.999 ± 0.053 | 1.051 ± 0.070 | 1.014 ± 0.110 | 0.962 ± 0.087 | 0.928 ± 0.066 |
|  | 14 | 0.974 ± 0.037 | 0.963 ± 0.064 | 0.981 ± 0.068 | 1.010 ± 0.120 | 0.807 ± 0.048 |
|  | 28 | 1.005 ± 0.035 | 0.949 ± 0.069 | 0.241 ± 0.076 | 0.461 ± 0.085 | 0.321 ± 0.100 |
|  | 42 | 1.223 ± 0.081 | 0.741 ± 0.130 | 0.157 ± 0.093 | 0.177 ± 0.127 | 0.123 ± 0.042 |
|  | 56 | 0.931 ± 0.081 | 0.307 ± 0.094 | 0.091 ± 0.020 | 0.110 ± 0.057 | 0.097 ± 0.027 |
| *M. hyopneumoniae-*specific IgG in serum measured with an in-house indirect ELISA (mean NetOD-values) | 0 | 0.067 ± 0.033 | 0.111 ± 0.087 | 0.075 ± 0.059 | 0.081 ± 0.041 | 0.075 ± 0.045 |
|  | 14 | 0.062 ± 0.043 | 0.135 ± 0.149 | 0.144 ± 0.145 | 0.108 ± 0.088 | 0.099 ± 0.046 |
|  | 28 | 0.066 ± 0.032 | 0.182 ± 0.287 | 0.326 ± 0.264 | 0.184 ± 0.139 | 0.271 ± 0.098 |
|  | 42 | 0.083 ± 0.033 | 0.249 ± 0.390 | 0.832 ± 0.261 | 0.606 ± 0.317 | 0.760 ± 0.219 |
|  | 56 | 0.127 ± 0.048 | 0.381 ± 0.390 | 1.346 ± 0.357 | 1.106 ± 0.509 | 1.135 ± 0.375 |
| *M. hyopneumoniae-*specific IgA in serum measured with an in-house indirect ELISA (mean NetOD-values ± SD) | 0 | 0.031 ± 0.034 | 0.020 ± 0.014 | 0.015 ± 0.007 | 0.019 ± 0.012 | 0.024 ± 0.031 |
|  | 14 | 0.032 ± 0.023 | 0.040 ± 0.028 | 0.035 ± 0.018 | 0.033 ± 0.020 | 0.031 ± 0.023 |
|  | 28 | 0.051 ± 0.045 | 0.039 ± 0.027 | 0.075 ± 0.051 | 0.050 ± 0.033 | 0.050 ± 0.023 |
|  | 42 | 0.044 ± 0.019 | 0.075 ± 0.057 | 0.260 ± 0.146 | 0.105 ± 0.048 | 0.155 ± 0.087 |
|  | 56 | 0.048 ± 0.027 | 0.167 ± 0.123 | 0.328 ± 0.170 | 0.243 ± 0.148 | 0.172 ± 0.074 |
| Number of *M. hyopneumoniae-*specific IgA positive animals in BAL fluid (% positive animals) determined with an in-house indirect ELISA | 42 | 0/5 (0.00) | 0/11 (0.00) | 9/11 (81.82) | 6/12 (50.00) | 5/12 (41.67) |
|  | 56 | 0/5 (0.00) | 11/11 (100.00) | 12/12 (100.00) | 12/12 (100.00) | 12/12 (100.00) |
| *M. hyopneumoniae-*specific IgA in BAL fluid measured with an in-house indirect ELISA (mean NetOD-values ± SD) | 42 | 0.069 ± 0.029 | 0.022 ± 0.029 | 0.519 ± 0.397 | 0.272 ± 0.291 | 0.185 ± 0.224 |
|  | 56 | 0.071 ± 0.059 | 0.640 ± 0.203 | 1.338 ± 0.400 | 0.858 ± 0.380 | 1.142 ± 0.363 |
